# Effects of ‘The Vicious Worm’ educational tool on *Taenia solium* knowledge retention in Zambian primary school students after one year

**DOI:** 10.1371/journal.pntd.0007336

**Published:** 2019-05-20

**Authors:** Emma C. Hobbs, Kabemba Evans Mwape, Brecht Devleesschauwer, Inge Van Damme, Meryam Krit, Dirk Berkvens, Gideon Zulu, Moses Mambwe, Mwelwa Chembensofu, Chiara Trevisan, Jacoba Baauw, Isaac Khozozo Phiri, Niko Speybroeck, Jennifer Ketzis, Pierre Dorny, Arve Lee Willingham, Sarah Gabriël

**Affiliations:** 1 Department of Biomedical Sciences, One Health Center for Zoonoses and Tropical Veterinary Medicine, Ross University School of Veterinary Medicine, Basseterre, St Kitts, West Indies; 2 Department of Biomedical Sciences, Institute of Tropical Medicine, Antwerp, Belgium; 3 Department of Veterinary Public Health and Food Safety, Faculty of Veterinary Medicine, Ghent University, Merelbeke, Belgium; 4 Department of Clinical Studies, School of Veterinary Medicine, University of Zambia, Lusaka, Zambia; 5 Department of Epidemiology and Public Health, Sciensano, Brussels, Belgium; 6 Department of Public Health, Ministry of Health, Government of the Republic of Zambia, Lusaka, Zambia; 7 Institute for Health Research and Society, Université catholique de Louvain, Institute of Health and Society (IRSS), Brussels, Belgium; University of Minnesota, UNITED STATES

## Abstract

**Background:**

*Taenia solium* is a neglected zoonotic parasite endemic throughout many low-income countries worldwide, including Zambia, where it causes human and pig diseases with high health and socioeconomic burdens. Lack of knowledge is a recognized risk factor, and consequently targeted health educational programs can decrease parasite transmission and disease occurrence in endemic areas. Preliminary assessment of the computer-based education program ‘The Vicious Worm’ in rural areas of eastern Zambia indicated that it was effective at increasing knowledge of *T*. *solium* in primary school students. The aim of this study was to evaluate the impact of ‘The Vicious Worm’ on knowledge retention by re-assessing the same primary school students one year after the initial education workshops.

**Methodology/Principal findings:**

Follow-up questionnaires were administered in the original three primary schools in eastern Zambia in 2017, 12 months after the original workshops. In total, 86 pupils participated in the follow-up sessions, representing 87% of the initial workshop respondents. Knowledge of *T*. *solium* at ‘follow-up’ was significantly higher than at the initial ‘pre’ questionnaire administered during the Vicious Worm workshop that took place one year earlier. While some specifics of the parasite’s life cycle were not completely understood, the key messages for disease prevention, such as the importance of hand washing and properly cooking pork, remained well understood by the students, even one year later.

**Conclusions/Significance:**

Results of this study indicate that ‘The Vicious Worm’ may be an effective tool for both short- and long-term *T*. *solium* education of rural primary school students in Zambia. Inclusion of educational workshops using ‘The Vicious Worm’ could be recommended for integrated cysticercosis control/elimination programs in sub-Saharan Africa, particularly if the content is simplified to focus on the key messages for prevention of disease transmission.

## Author summary

The zoonotic parasite *Taenia solium*, commonly known as the pork tapeworm, causes substantial public health and economic losses worldwide. It is commonly found in low-income countries where pigs are raised in areas of poor sanitation, including Zambia. The links between the parasite and its different disease forms in humans and pigs are not very well known, and ignorance of the parasite is a known risk factor for infection. Health education can significantly increase knowledge and awareness of the parasite and can inspire behavioral change that reduces disease transmission. ‘The Vicious Worm’ is a computer-based program designed to provide *T*. *solium* education in a fun and interactive way. We conducted educational workshops in three primary schools in rural areas of eastern Zambia, and preliminary assessment indicated that the ‘Vicious Worm’ educational content significantly improved students’ knowledge of *T*. *solium*. We also conducted follow-up studies in the same students one year later, and discovered that the students’ knowledge was still significantly higher than at baseline. We conclude that ‘The Vicious Worm’ may be a useful educational component to enable targeting of school students, and would recommend its inclusion in integrated *T*. *solium* control programs in future.

## Introduction

*Taenia solium* is a zoonotic parasite known as the pork tapeworm, which infects over 50 million people worldwide [1]. Invasion of the human brain by the larval stage of the parasite is known as neurocysticercosis (NCC), which can cause neurological deficits including severe progressive headache, stroke and hydrocephalus, and is the world’s leading cause of preventable epilepsy [2]. Other impacts of human infection include treatment costs, productivity losses and social stigmatization of epilepsy sufferers [3]. Porcine infections (porcine cysticercosis, PCC) cause substantial economic losses from carcass condemnation, and reductions to farmer income and food safety that exacerbate the poverty cycle in many developing countries in which the parasite is endemic [4, 5].

Despite global ‘tool readiness’ for control of *T*. *solium* [6], high levels of active parasite transmission persist in many endemic countries throughout Latin America, Asia and sub-Saharan Africa, including Zambia. Transmission is to a large extent socially determined, with inadequate sanitation, poor hygiene practices, minimal access to medical or veterinary services, and low levels of health education enabling parasite transmission in areas where pigs are raised. A lack of knowledge of the parasite has been identified as one of the barriers for control, and targeted health education interventions have been shown to be an effective addition to other *T*. *solium* control measures [7–11]. Education is recognized by the World Health Organization as an important part of the multisectoral approach needed for control of zoonotic pathogens such as *T*. *solium* [12]. Computer-based tools have the advantages of providing standardized educational messages, reduce training costs, are able to be widely disseminated and can be updated more easily, compared to traditional paper-based learning systems [13].

‘The Vicious Worm’ (https://theviciousworm.sites.ku.dk) is a freely-downloadable computer-based educational program designed to provide comprehensive information about *T*. *solium* in a fun and interactive way. It is set in a sub-Saharan African context and has different levels of detail to allow tailoring of the educational content to suit the needs of the target audience [13]. Studies with medical and agricultural professionals in Tanzania demonstrated significant knowledge uptake and retention, and reported behavioral changes and knowledge dissemination directly attributable to exposure to ‘The Vicious Worm’ [14, 15]. The program had not previously been evaluated for use in school-going children, who have been shown to be effective ‘health change agents’ capable of effectively disseminating educational messages to family and community members [16, 17]. A preliminary study conducted by the authors of this manuscript in three primary schools in the highly *T*. *solium*–endemic Eastern Province of Zambia in 2016 demonstrated significant uptake of *T*. *solium*-associated knowledge in adolescent primary school pupils in the short-term [18]. The study at hand revisited the same primary school pupils one year later, to evaluate the longer-term impact of ‘The Vicious Worm’ on *T*. *solium*–associated knowledge retention.

## Methods

### Study area

The study took place in the Nyembe (Katete district), Chimvira and Herode (Sinda district) communities in the Eastern Province of Zambia. As discussed in [18], the region is highly endemic for *T*. *solium*; prevalence of active human and pig infections are among the highest in the world, and over 57% of human epilepsy cases are attributable to NCC [19, 20].

### Study design

**Fig 1:**
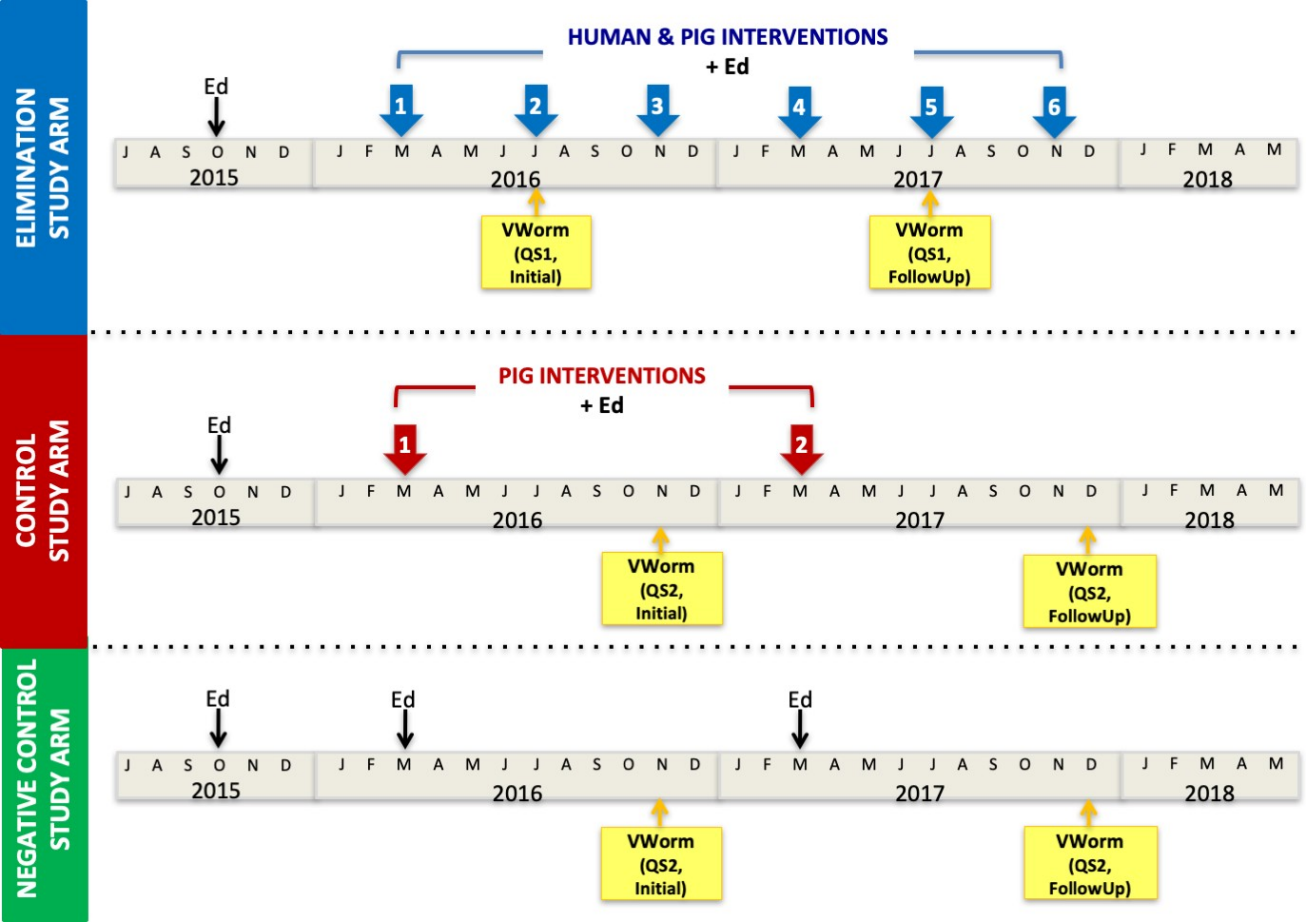
Timeline of CYSTISTOP’s interventional and educational activities in the three study arms. Village-based educational sessions (Ed) were conducted in all three study arms at baseline, then every four months in the elimination study arm, and annually in the control study arm in conjuction with intervention activities. Education alone was conducted annually in the negative control study arm. Initial Vicious Worm workshops (VWorm) were conducted in one primary school in each of the three study arms in 2016, and follow-up sessions were conducted one year later. Assessment questionnaire 1 (QS1) was used in the elimination study arm workshops, while the revised QS2 was used in the control and negative control study arms.

‘CYSTISTOP’ is a prospective, large-scale community-based *T*. *solium* intervention study, which commenced in three study arms in the Katete and Sinda districts in the Eastern Province of Zambia in 2015. The study has two intervention arms designed to compare integrated human- and pig-based interventions (elimination study arm) versus pig-only (control study arm) interventions, as compared to a negative control study arm. Health education was also conducted at four- (elimination study arm) and twelve-monthly (control and negative control study arms) intervals (Fig 1). Health educational methods included village-based educational sessions during sensitization, conducted in Chewa (the local language) by a trained bilingual CYSTISTOP program member. These sessions included descriptions of the parasite’s life cycle and ways to prevent its transmission in the villages, and utilized visual aids including a large canvas life cycle poster, a five-meter long ribbon to represent the adult tapeworm, and life-sized plasticine models of human stool demonstrating expelled tapeworm proglottids. Participation in village-based sensitization sessions was higher in the elimination study arm than in the control study arm (89% compared to 46%, [35]), and sessions were primarily attended by women, very young children, and few men (personal observation.)

Large color posters of the parasite’s life cycle were permanently displayed at the rural health centers in each of the three study areas. Simplified A4-sized paper copies of the life cycle poster were also distributed to each household in the two intervention study areas (elimination and control study arms) during the baseline visits in October 2015. The final component of CYSTISTOP’s health education intervention was workshops in primary schools using the ‘The Vicious Worm’ computer program.

**Fig 2:**
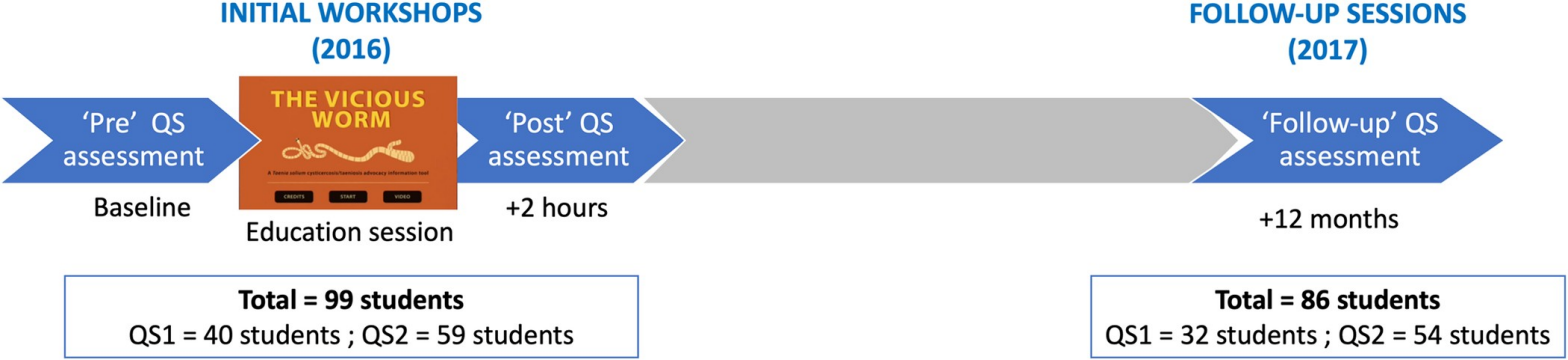
Timeline of ‘The Vicious Worm’ educational sessions in the three study arms. Initial workshops in 2016 consisted of a 'pre' questionnaire (QS), an educational session using ‘The Vicious Worm’, and a 'post' QS immediately after. The 'follow-up' QS sessions were conducted one year later. QS1 was used in the elimination study arm; QS2 was used in the control and negative control study arms.

The educational workshops were conducted in Nyembe (elimination study arm) in July 2016, and in the Kondwelani (control study arm) and Gunda (negative control study arm) primary schools in November 2016 as described in [18]. The initial workshops comprised a ‘pre’ questionnaire to assess baseline knowledge, an educational session using ‘The Vicious Worm’, followed immediately by a ‘post’ questionnaire to evaluate knowledge uptake (see Fig 2). Follow-up sessions were scheduled in the same primary schools in July (elimination study arm) and early December (control and negative control study arms) 2017, one year after the initial workshops.

There were two questionnaires (QS) used in the sessions as per Hobbs et al [18]: the original questionnaire (QS1), modified from the original questionnaire [14] to include Zambian terminology, was used in the elimination study arm and had 24 questions grouped into eight categories. As the QS was deemed too long and complicated for primary school pupils, a simplified version (QS2) containing 15 questions in three categories was subsequently used in the control and negative control study arms. Both QS were designed to test knowledge of human tapeworm infections, known as taeniosis (TS); human (neuro)cysticercosis (NCC/CC); and PCC, including the linkages between the disease states and methods of transmission, diagnosis, and prevention. (The QS used in the sessions are provided in the data repository.)

### Follow-up sessions

All of the pupils who had attended the initial educational workshops in 2016 were invited to return for a follow-up session, conducted in the same primary schools in July (elimination study arm) and December (control and negative control study arms) 2017.

Follow-up sessions were conducted as per the ‘post’ QS used in the initial workshops, as described in [18], and were conducted by one of the same two trained bilingual CYSTISTOP project members as in the original workshops. Briefly, QS were projected onto a classroom wall, and questions and answer options were read aloud in Chewa and repeated at least once for clarity. Using Bluetooth-connected TurningPoint clicker devices, all pupils had to individually submit their answer to each question before the group could proceed to the next question. At the conclusion of each session, the group was taken through the QS again to discuss the correct answers and address any remaining misconceptions. The sessions were between 30 (QS2) and 45 (QS1) minutes in duration.

### Data management and statistics

The differences in the two questionnaires prevented direct comparison of response data, so QS1 data (elimination study arm) were analyzed separately from QS2 (control and negative control study arms). Each question was scored as either correct (1) or incorrect (0), resulting in a maximum score of 24 for QS1 and 15 for QS2. Some questions in QS1 had more than one correct answer; selection of any one of these answers resulted in a ‘correct’ outcome. Group (QS1 and QS2) and individual (QS2 only; a technical problem prevented the collection of individual QS1 data during the initial elimination study arm workshop) responses to each session were exported into an Excel (Microsoft Corporation, 2010) spreadsheet for descriptive statistics. Responses were assessed individually and by category.

Grouped result data for QS1 were analyzed using a generalized linear model, using the number of positive and negative answers as binomial response variable, and study time point as categorical covariate. The absence of individual data did not allow taking the within-respondent correlations across study time points into account. Pairwise comparisons of mean scores by study time point were performed using Tukey’s all-pair comparisons method.

Individual result data for QS2 collected at both baseline and follow-up allowed further analyses. The analysis of the correlated ‘pre’, ‘post’ and ‘follow-up’ scores was carried out using a generalized linear mixed model using individual respondent as random effect, the number of positive and negative answers as binomial response variables, and study time point as categorical covariate. Pairwise comparisons of mean scores by study time point were performed using Tukey’s all-pair comparisons method. Additional multivariable analyses were performed adding the respondents’ age, gender, and school. This model was applied to the total scores and to each of the three categories. The analyses were performed using the lme4 and multcomp packages for R 3.5.1 [21–23].

### Ethics statement

This study was conducted as part of the ongoing CYSTISTOP project (https://clinicaltrials.gov/ct2/show/NCT02612896). Ethical clearance was obtained from the University of Zambia Biomedical Research Ethics Committee (004-09-15) and the Ethical Committee of the University of Antwerp, Belgium (B300201628043, EC UZA16/8/73). The study was introduced and explained to all project participants, both in village group settings and within individual households, prior to each field visit. Written informed consent to participate in the workshops, voluntarily provided by a parent or guardian, was obtained for each pupil, and attendance at the educational sessions was voluntary. The sessions took place outside of normal school hours. There was no incentive for participation, but light refreshments were provided after the sessions.

## Results

A total of 86 pupils participated in the follow-up sessions, of whom 55% were female. Ages ranged from 10–18 years, with a median of 14 years. QS1 was taken by 32 of the original 40 pupils whereas QS2 was taken by 54 of the 59 original pupils (83% and 92% follow-up rates, respectively).

Individual analyses of QS2 data revealed that there were no significant differences based on age, gender or village (control study arm vs negative control study arm). Consequently, QS2 data are presented as a consolidated dataset.

### QS1 results

#### Results of follow-up assessment by question

Proportions of the group answering a question correctly ranged from 41% to 100%, with an average correct proportion of 76% (see S1 File). Of the 24 questions, 20 (83%) were answered correctly by more than 50% of the group, and 16 (67%) were correctly answered by 75% or more of the group. The whole group correctly answered, ‘What are the symptoms of NCC?’

Of the four questions answered (technically–see discussion) incorrectly by more than half the group, two were related to management of live and slaughtered pigs that were found to be infected with PCC, and in both instances the majority of the group selected the options to destroy the pig.

#### Results of follow-up assessment by category

The category scores ranged from 42–90%, with seven of the eight categories (88%) answered correctly by between at least half the group and five (63%) by at least 80% of the group (see Table 1). The least understood category was ‘PCC treatment’, followed by ‘Relationship between PCC/TS/NCC’.

**Table 1:** Results from ‘pre’, ‘post’ and ‘follow-up’ assessments (QS1).

	INTIAL WORKSHOP			FOLLOW-UP SESSION				
	Correct during 'pre' (%)	Correct during 'post' (%)	Knowledge change (%)	Correct during 'follow-up' (%)	Knowledge change (%): 'Follow up' vs 'post'	P-value for difference: 'Follow up' vs 'post'	Knowledge change (%): 'Follow up' vs 'pre'	P-value for difference: 'Follow up' vs 'pre'
Category 1: Acquisition & transmission of *T*. *solium* infections	65.3	85.0	19.7	82.3	-2.7	0.853	17.0	0.015
Category 2: Acquisition of NCC	26.5	20.5	-6.0	53.1	32.6	<0.001	26.6	0.003
Category 3: TS in general	74.3	85.3	11.0	89.6	4.3	0.580	15.3	0.015
Category 4: NCC in general	69.3	87.0	17.7	88.5	1.5	0.909	19.2	0.003
Category 5: PCC diagnosis	68.8	83.8	15.0	89.8	6.1	0.293	21.1	<0.001
Category 6: PCC treatment	35.0	41.5	6.5	42.2	0.7	0.993	7.2	0.652
Category 7: Relationship between PCC/TS/NCC	61.0	67.7	6.7	54.2	-13.5	0.114	-6.8	0.586
Category 8: Prevention of PCC/TS/NCC	70.3	85.3	15.0	81.3	-4.1	0.668	10.9	0.143
**OVERALL QUESTIONNAIRE AVERAGES**	**62.0**	**73.5**	**11.5**	**76.0**	**2.5**	**0.404**	**14.0**	**<0.001**

#### Knowledge retention by question

In total, 18 (75%) of the 24 questions were answered more successfully during the follow-up sessions compared to the ‘pre’ QS, and 14 (58%) more successfully than in the ‘post’ QS. Increases of 10% or more were seen in 16 questions (67%), and 6 (25%) increased by at least 25% compared to ‘post’ results.

The average percentage of correct answers for QS1 was 76%, an increase from both the ‘pre’ (62%, P<0.001) and ‘post’ (74%, P = 0.293) rounds. The number of questions that were correctly answered by at least 75% of the group remained constant at 18 (67%), however the number that were answered correctly by 90% or more of the group increased from six in the ‘post’ QS to eight in the follow-up QS.

Compared to the ‘pre’ QS data, four (17%) questions at ‘follow-up’ showed decreased knowledge. The most substantial decrease was for the question, ‘Is PCC a problem for human health?’, which had decreased from 88% at both ‘pre’ and ‘post’ rounds to 56% at ‘follow-up’, with 24% of respondents at follow-up selecting the incorrect response, ‘Yes, humans can get neurological symptoms by eating infected pork.”

#### Knowledge retention by category

Knowledge by category was not significantly different between ‘post’ and ‘follow-up’ stages, with 80% (6/8) of category averages at ‘follow up’ within 10% of ‘post’ values.

The category that showed the most improvement was ‘Acquisition of NCC’, which increased by 33% since the ‘post’ round and 27% since the ‘pre’ round. However, with an average category score of 53% at ‘follow-up’, this remained one of the less successfully answered categories.

The average group score for the ‘Relationship between PCC/TS/NCC’ category decreased the most, from 61% at ‘pre’ and 68% at ‘post’, to 54% at ‘follow-up’. This was largely due to the 32% decrease in correct answers for the question, ‘Is PCC a problem for human health?’ as discussed above.

### QS2 results

#### Results of follow-up assessment by question

The average question score for the follow-up QS was 71%, with individual question scores ranging from 28–93% ((see S2 Table). Of the 15 questions, 12 (80%) were answered correctly by at least half of respondents, while nine questions (60%) were answered correctly by at least three-quarters of pupils.

Of the two questions answered incorrectly by more than half the pupils, the question ‘How do people become infected with CC?’ was answered the most poorly. Only 28% of respondents answered this question correctly, while the rest (72%) selected the answer ‘By eating undercooked pork infected with *T*. *solium*’. The question, ‘Can people with NCC transmit it to others?’ was answered correctly by 48% of pupils, with 41% selecting the incorrect response, ‘Yes, by coughing/sneezing’.

#### Results of follow-up assessment by category

The ‘Prevention’ category was the most successfully answered, with an average score of 78%. The other categories were answered correctly by 76% (‘General knowledge’) and 60% (‘Transmission’) of the individuals (see Table 2).

**Table 2:** Results from ‘pre’, ‘post’ and ‘follow-up’ assessments (QS2).

	INITIAL WORKSHOP			FOLLOW-UP SESSION				
	Correct during 'pre' (%)	Correct during 'post' (%)	Knowledge change (%)	Correct during 'follow up' (%)	Knowledge change (%): 'Follow up' vs 'Post'	P-value for difference: 'Follow up' vs 'post'	Knowledge change (%): 'Follow up' vs 'Pre'	P-value for difference: 'Follow up' vs 'pre'
Category 1: General knowledge	70.8	90.8	20.0	75.9	-14.9	<0.001	5.1	0.105
Category 2: Transmission	48.1	68.8	20.7	59.6	-9.2	0.058	11.5	0.035
Category 3: Prevention	65.8	86.4	20.7	78.1	-8.3	0.020	12.3	0.013
**OVERALL QUESTIONNAIRE AVERAGES**	**61.6**	**82.0**	**20.4**	**71.2**	**-10.8**	**<0.001**	**9.6**	**<0.001**

#### Knowledge retention by question

**Fig 3:**
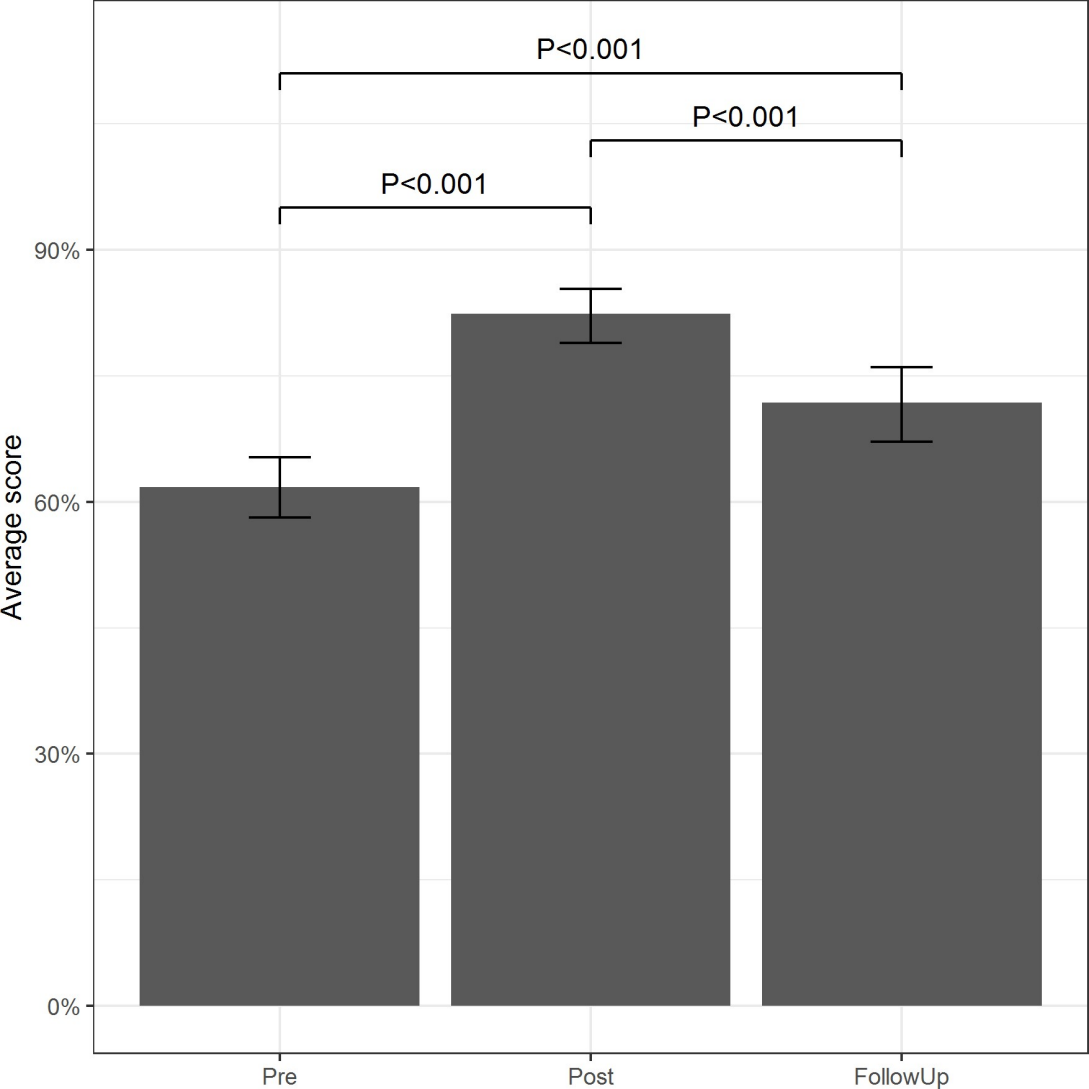
Average individual pupil scores for QS2 at each of the three assessment points. Statistical significance between time points is indicated by the P-values.

Average individual scores for QS2 at ‘pre’, ‘post’ and ‘follow-up’ assessments are presented in Fig 3. Compared to ‘pre’ data, the average score for the follow-up round increased by 10% (P<0.001), with the proportion of questions correctly answered by at least three-quarters of the group nearly doubling from five at baseline (33%) to nine at follow-up (60%).

When comparing ‘follow-up’ with ‘post’ data however, there was an 11% decrease in the average score (P<0.001), with 80% of questions answered less successfully during ‘follow-up’ compared to the year prior.

The question, ‘How can a pig become infected with PCC?’ showed the biggest decrease in knowledge, from 92% at ‘post’ compared to 68% at ‘follow-up’. This was due to more respondents at this time selecting the answers, ‘By being mated with an infected pig’ (17%) and ‘By eating maize bran or rice that has gone moldy’ (11%).

#### Knowledge retention by category

Increases were seen in all three categories from ‘pre’ to ‘follow-up’ rounds, with statistically significant increases of at least 10% in the ‘Transmission’ (P = 0.035) and ‘Prevention’ categories (P = 0.013).

Compared to ‘post’ data however, all three category scores at ‘follow-up’ had decreased (P≤0.06). The ‘Prevention’ category had decreased the least, while the ‘General knowledge’ category decreased the most.

## Discussion

This follow-up study indicates that educational workshops using ‘The Vicious Worm’ may have lasting positive effects on *T*. *solium* knowledge uptake and retention in rural adolescent primary school pupils in eastern Zambia. Knowledge levels at ‘follow-up’ were significantly higher than at baseline one year earlier, with increases of 14% and 10% compared to ‘pre’ levels in QS1 and QS2, respectively. Compared to ‘post’ knowledge levels immediately following the educational component one year earlier, however, knowledge at ‘follow up’ was similar (QS1) or significantly lower (QS2). The questions relating to general knowledge of TS and NCC, diagnosis of PCC, and prevention of PCC/TS/NCC were answered very well in both QS at ‘follow-up’, with 63% of categories in QS1 and 66% of categories in QS2 answered correctly by at least 75% of the groups. The knowledge regarding prevention of the parasite’s transmission was both the best answered category, and showed the lowest decrease in knowledge from the ‘post’ round one year earlier. This indicates that although some aspects of the parasite’s life cycle remained imperfectly understood at ‘follow-up’, the pupils generally retained the main aspects of *T*. *solium* and the key messages for disease prevention one year after ‘The Vicious Worm’ educational workshops.

The parasite’s life cycle is complex, and certain aspects remained imperfectly understood by the pupils at ‘follow-up’. Transmission of PCC was not well understood, nor was transmission of NCC/CC in humans. Many respondents from both QS selected the incorrect answer responses stating that NCC/CC is obtained via ingestion of raw or undercooked pork that is infected with PCC, which given the complexity of the *T*. *solium* life cycle is not surprising. Indeed, many other field studies have demonstrated similar results with adults, farmers and even veterinary and medical professionals showing imperfect understanding of the life cycle despite educational interventions [7, 9, 10, 14, 17, 24]. However, what is of concern from these data is that some respondents apparently believed that people with NCC/CC or specifically epilepsy can transmit the disease to others (24%, QS2). Epilepsy is often stigmatized in many low-income countries including Zambia, and the social and psychological effects of stigmatization can substantially decrease quality of life for epilepsy sufferers and their families [25, 26]. While the majority of other respondents correctly indicated that NCC is not transmissible to others, this message should be particularly emphasized in future educational interventions.

Many pupils again selected destruction of the pig and/or carcass as the most suitable method for management of live or slaughtered pigs with PCC, as was also seen in the initial workshops and discussed in [18]. While the ‘correct’ answers for the purposes of the QS scoring were treating pigs with oxfendazole or properly cooking pork, destruction of and proper disposal of heavily infected pork is in fact the recommended approach mandated by World Organization for Animal Health’s (OIE) Terrestrial Animal Health Code [27] and the Zambian Public Health Act [28], and this should be reflected in the marking of these questions in future workshops. However, the OIE Terrestrial Animal Health Code also states that the meat of carcasses infected with less than 20 cysticerci can be consumed after treatment (that is, freeze- or heat-treatment, with the latter reaching a core temperature of 80°C). As ‘backyard’ animal slaughter is frequently conducted in rural and remote communities in many developing countries including Zambia, meat inspection is often rudimentary or absent. Given the limited availability of nutrition and particularly protein in many rural and remote developing communities, insisting on strict measures pertaining to meat inspection and condemnation is not always realistic, and may foster resistance and/or resentment in some situations. We therefore feel it is important to also highlight the alternative options to carcass destruction, especially considering the nutritional needs of these and many other low-resource communities that are endemic for *T*. *solium*. Consequently, we would recommend that future educational messages and workshops should recommend destruction of heavily infected meat and carcasses wherever possible, while also promoting proper cooking of lightly infected meat and/or anthelmintic treatment of pigs as more realistic alternatives for some resource-poor endemic communities.

The reason for the decreased knowledge regarding PCC transmission routes seen in students from the control and negative control study arms at ‘follow-up’ (more students indicating that infection arises after pigs being mated with an infected pig, or after eating moldy feedstuff) is unclear, but may be related to the decreased frequency of educational delivery in these study arms compared to in the elimination study arm.

Adolescent primary school pupils were selected to participate in these educational workshops because studies have shown that school students can be ‘health change agents’ capable of effectively disseminating educational messages to family and community members [16, 17]. A cluster-based education trial in northern Tanzania utilized leaflets and videos containing *T*. *solium*-specific health education in primary and secondary schools, and demonstrated generally increased knowledge and attitudes in pupils from intervention schools compared to control schools [17].

Using computer-based programs allows standardization of educational messages, while allowing flexibility and adaptation of the content to specific audiences. The recent release of ‘The Vicious Worm’ as a multiplatform smartphone app and the completed translation of the online version into Kiswahili [29], will allow expansion of the program across the African continent. Other language translations are currently underway (personal communication, C. Trevisan), and with adaptation of the illustrations and contexts for Latin American, Asian or other specific settings, this tool could be implemented worldwide. Other electronic educational media including short animated videos, talking books, songs and DVDs are increasingly used in public health campaigns around the world, with encouraging results [30]. In a Chinese study, a short animated cartoon called ‘The Magic Glasses’ was shown to halve infection rates of parasitic worms in school-aged children (8.4–4.1%, P<0.0001), and observed occurrence of handwashing increased from 54% to 98.9% (P<0.0001) in the intervention group compared to the control group [31]. Tablet-based educational interventions have also been successful at raising awareness and changing behaviors for prevention of other, non-parasitic diseases, including cervical cancer and human papilloma-virus infections [32].

It should be emphasized that increased knowledge and awareness of a topic does not necessarily translate into behavioral change, and there may be underlying sociocultural and/or economic factors contributing to parasite transmission in endemic communities that can override even known adverse health outcomes associated with certain behaviors [33, 34]. Student responses given during these assessment situations may indicate what the students believed to be technically correct answers, rather than reflecting their actual behaviors and beliefs. Feedback from focus group discussions conducted in the elimination and control study arms indicated that behavioral changes have been initiated in the villages since the start of the CYSTISTOP project [35], and follow-up observational visits to the study areas are planned for 2019 to corroborate these reports.

The effectiveness of information transfer from educated individuals to others is difficult to quantify, and evaluation of such knowledge transfer was not within the scope of this study. A primary school-based health education trial in Tanzania demonstrated significant knowledge uptake in pupils from intervention schools compared to control schools, whereas evaluation of knowledge transfer to the community showed mixed results [36]: some parents reportedly implemented behavioral changes such as building toilets and boiling drinking water based on knowledge passed on from their children; others reportedly wished to do more but lacked resources to do so; and some parents found it improper for children to instruct their parents. Mwidunda et al [17] reported that secondary school students are often more respected in their families and communities than primary school pupils, and suggested that focusing health educational messages on secondary schools may increase effects of knowledge transfer to communities. No secondary schools are present in the study areas, as is typically the case for many remote and rural regions of Zambia, but conducting Vicious Worm workshops in secondary schools would be encouraged where possible.

This study has limitations. The project activities including health education were conducted more frequently in the elimination study arm (four-monthly) than in the control and negative control study arms (annually), which could have been at least partially responsible for the seemingly better knowledge retention at ‘follow-up’ demonstrated by the elimination study arm students (QS1). The use of two different QS prevented direct comparison of knowledge uptake and retention from individuals across all three study arms, which would have allowed even more robust analyses. In addition, because the technical error in the initial elimination study arm workshop prevented collection of individual response data, we only had grouped result data for QS1, and were consequently not able to take the within-respondent correlation across study time points into account. This led to an underestimation of variances, and consequently an increased probability of (falsely) detecting significant associations. The comparisons across study time points for QS1 should therefore be interpreted with caution. The loss of twelve of the original students to follow-up in this study is another limitation, however statistical significance was nevertheless achieved. Evaluating the effects of knowledge uptake on behavioral change or the extent of knowledge transfer from students to others was outside the scope of this study, but would be useful to attempt in future studies.

In future educational workshops using ‘The Vicious Worm’ it may be beneficial, as per the authors’ previous recommendations [18], to modify the educational component to focus on the main methods for prevention of disease transmission, rather than detailing the *T*. *solium* life cycle. Tailoring educational materials to the specific sociocultural context, including use of non-textual media to include individuals with low literacy skills, may further enhance education uptake in endemic communities. The use of locally-broadcast radio programs or simple, illustrative printed material such as posters, leaflets and comic books may also add value to educational programs [7, 8, 11, 37], especially in areas where access to smartphones or computers is limited. Some standardized educational posters are available for *T*. *solium* education [38], including several recently published online by the European Network on Taeniosis/Cysticercosis (CYSTINET, COST Action TD1302, http://www.cystinet.org/) (see **S1 File**).

The results from this follow-up study demonstrate that educational workshops using ‘The Vicious Worm’ can contribute to significantly increased *T*. *solium* knowledge in rural Zambian primary school students in both the short- and long-term. Despite some confusion regarding the precise relationships between TS, NCC/CC and PCC, in general the data indicate that the key messages for prevention of disease transmission, including the importance of hand washing and of proper cooking of pork, remained well understood by the students one year after the educational sessions. The flexible nature of ‘The Vicious Worm’ program, combined with recent and ongoing translations into languages other than English and the development of the app for smartphones, provides standardized educational content that can be tailored to the specific educational and sociocultural context of the target audience. For village-level educational interventions in rural endemic communities it may be advised to simplify or omit the more scientific aspects of ‘The Vicious Worm’ in favor of promoting key behavioral messages, to enhance knowledge uptake and retention. Focusing education on school-going children as key change agents may also increase community awareness and engagement. Tailored ‘Vicious Worm’-based educational interventions should be considered for incorporation with integrated *T*. *solium* control or elimination programs in future.

## Supporting information

S1 Table Complete results from ‘pre’, ‘post’ and ‘follow-up’ assessments (QS1). ^a^ '*Masese*' is the local (Chewa language) word for CC. ND = not done.
(DOCX)

S2 Table Complete results from ‘pre’, ‘post’ and ‘follow-up’ assessments (QS2). ^a^ '*Masese*' is the local (Chewa language) word for CC. ND = not done.
(DOCX)

S1 File **Educational posters: ‘5 things to do about the pork tapeworm (*Taenia solium)*’, in English (a) and Chewa (b).** Developed by CYSTINET (http://www.cystinet.org/). Illustrations by F. Jansen.
(DOCX)

## References

[1] WHO. WHO estimates of the global burden of foodborne diseases: foodborne disease burden epidemiology reference group 2007–2015. World Health Organization, 2015.

[2] WHO. Investing to overcome the global impact of neglected tropical diseases: third WHO report on neglected tropical diseases. Geneva, Switzerland: World Health Organization, 2015.

[3] Winkler AS. Neurocysticercosis in sub-Saharan Africa: a review of prevalence, clinical characteristics, diagnosis, and management. Pathog Glob Health. 2012;106(5):261–74. 10.1179/2047773212Y.0000000047 23265550 PMC4005109

[4] Hobbs EC, Mwape KE, Devleesschauwer B, Gabriël S, Chembensofu M, Mambwe M, et al *Taenia solium* from a community perspective: preliminary costing data in the Katete and Sinda districts in Eastern Zambia. Vet Parasitol. 2018;251:63–7. 10.1016/j.vetpar.2018.01.001 29426478

[5] Trevisan C, Praet N, Pondja A, Assane Y, Dorny P, Magnussen P, et al. Assessment of the social burden of Taenia solium cysticercosis in Angonia district, Mozambique. Poster session presented at 8th European Congress on Tropical Medicine and International Health and 5th Conference of the Scandinavian-Baltic Society for Parasitology, Copenhagen, Denmark.2013.

[6] Thomas LF. WHO landscape analysis—control of *Taenia solium*. Harare, Zimbabwe: World Health Organisation (WHO); 2015.

[7] Alexander AM, Mohan VR, Muliyil J, Dorny P, Rajshekhar V. Changes in knowledge and practices related to taeniasis/cysticercosis after health education in a south Indian community. Int Health. 2012;4(2012):164–9. 24029395 10.1016/j.inhe.2012.04.003

[8] Ngowi HA, Carabin H, Kassuku AA, Mlozi MRS, Mlangwa JED, Willingham AL III. A health-education intervention trial to reduce porcine cysticercosis in Mbulu District, Tanzania. Prev Vet Med. 2008;85(2008):52–67. 10.1016/j.prevetmed.2007.12.014 18243375

[9] Sarti E, Flisser A, Schantz PM, Gleizer M, Loya M, Plancarte A, et al Development and evaluation of a health education intervention against *Taenia solium* in a rural community in Mexico. Am J Trop Med Hyg. 1997;56(2):127–32. 9080868 10.4269/ajtmh.1997.56.127

[10] Wohlgemut J, Dewey C, Levy M, Mutua F. Evaluating the efficacy of teaching methods regarding prevention of human epilepsy caused by *Taenia solium* neurocysticercosis in western Kenya. Am J Trop Med Hyg. 2010;82(4):634–42. 10.4269/ajtmh.2010.09-0404 PMC2844555. 20348512 PMC2844555

[11] Carabin H, Millogo A, Ngowi HA, Bauer C, Dermauw V, Koné AC, et al Effectiveness of a community-based educational programme in reducing the cumulative incidence and prevalence of human *Taenia solium* cysticercosis in Burkina Faso in 2011–14 (EFECAB): a cluster-randomised controlled trial. Lancet Glob Health. 2018;6(4):e411–e25. 10.1016/S2214-109X(18)30027-5 29530423 PMC5873982

[12] WHO. Integrated control of neglected zoonotic diseases in Africa: applying the "One Health" concept France: World Health Organization [WHO], Department of Food Safety Z, and Foodborne Diseases; 2009.

[13] Johansen MV, Trevisan C, Braae UC, Magnussen P, Ertel RL, Mejer H, et al The Vicious Worm: a computer-based *Taenia solium* education tool. Trends Parasitol. 2014;30(8):372–4. 10.1016/j.pt.2014.06.003 25017127

[14] Ertel RL, Braae UC, Ngowi HA, Johansen MV. Assessment of a computer-based *Taenia solium* health education tool 'The Vicious Worm' on knowledge uptake among professionals and their attitudes towards the program. Acta Trop. 2017;165:240–5. Epub 1 Nov 2015. 10.1016/j.actatropica.2015.10.022 26536396

[15] Lauridsen S. Prevention of *Taenia solium* through electronic health education: An evaluation of learning outcome and changed practices one year after introduction to the computer-based tool 'The Vicious Worm' [MSc]. Copenhagen, Denmark: University of Copenhagen; 2016.

[16] Mwanga JR, Jensen BB, Magnussen P, Aagard-Hansen J. School children as health change agents in Magu, Tanzania: a feasibility study. Health Promot Int. 2007;23(1):8 10.1093/heapro/dam037 18086688

[17] Mwidunda SA, Carabin H, Matuja WBM, Winkler AS, Ngowi HA. A school based cluster randomised health education intervention trial for improving knowledge and attitudes related to *Taenia solium* cysticercosis and taeniasis in Mbulu district, northern Tanzania. PLOS One. 2015;10(2):e0118541 10.1371/journal.pone.0118541 25719902 PMC4342010

[18] Hobbs EC, Mwape KE, Van Damme I, Berkvens D, Zulu G, Mambwe M, et al Preliminary assessment of the computer-based *Taenia solium* educational program ‘The Vicious Worm’ on knowledge uptake in primary school students in rural areas in eastern Zambia. Trop Med Int Health. 2018;23(3):306–14. 10.1111/tmi.13029 29314480 PMC5888122

[19] Mwape KE, Blocher J, Wiefek J, Schmidt K, Dorny P, Praet N, et al Prevalence of neurocysticercosis in people with epilepsy in the Eastern Province of Zambia. PLOS Negl Trop Dis. 2015;9(8). 10.1371/journal.pntd.0003972 26285031 PMC4540454

[20] Mwape KE, Phiri IK, Praet N, Muma JB, Zulu G, Van den Bossche P, et al *Taenia solium* infections in a rural area of eastern Zambia-A community based study. PLOS Negl Trop Dis. 2012;6(3):e1594 10.1371/journal.pntd.0001594 22479664 PMC3313923

[21] Bates D, Mächler M, Bolker B, Walker S. Fitting linear mixed-effects models using lme4. J Stat Softw. 2015;67(1). 10.18637/jss.v067.i01

[22] Hothorn T, Bretz F, Westfall P. Simultaneous inference in general parametric models. Biom J. 2008;50(3):346–63. Epub 2008/05/16. 10.1002/bimj.200810425 . 18481363

[23] R Development Core Team. R: A language and environment for statistical computing Vienna, Austria: The R Foundation for Statistical Computing; 2011 Available from: http://www.r-project.org/].

[24] Ngowi H, Mkupasi EM, Lekule FP, Willingham AL III, Thamsborg SM. Impact of farmer education on their knowledge, attitudes and practices in southern Tanzania: a case for *Taenia solium* control. Livestock Research for Rural Development 2011.

[25] Riasi H, Sanati AR, Kazem G. The stigma of epilepsy and its effects on marital status. SpringerPlus. 2014;3:762 10.1186/2193-1801-3-762 PMC4320190. 25674487 PMC4320190

[26] WHO. Global burden of epilepsy and the need for coordinated action at the country level to address its health, social and public knowledge implications: Report by the Secretariat; Sixty-eighth World Health Assembly. Geneva, Switzerland: WHO, 2015 27 3 2015. Report No.: Contract No.: A68/12, Provisional agenda item 13.5.

[27] OIE. Infection with *Taenia solium* (porcine cysticercosis) 2016 In: Terrestrial Animal Health Code [Internet]. World Organization for Animal Health.

[28] Public Health Act (Cap. 295), (1930 (2006)).

[29] Trevisan C, Fèvre EM, Owiny M, Ngere I, Vang Johansen M. Minyoo Matata–The Vicious Worm–a *Taenia solium* computer-based health-education tool–in Swahili. Trends Parasitol. 2017;33(10):746–8. 10.1016/j.pt.2017.05.012 28634004 PMC5636612

[30] Hobbs EC, Trevisan C, Johansen MV, Dorny P, Gabriel S. Value of electronic educational media in combatting parasitic diseases. Trends Parasitol. 2018;Epub ahead of print, 22 October 2018. Epub 22 October 2018. 10.1016/j.pt.2018.10.001. 30360957

[31] Bieri FA, Gray DJ, Williams GM, Raso G, Li Y-S, Yuan L, et al Health-education package to prevent worm infections in Chinese schoolchildren. New England Journal of Medicine. 2013;368(17):1603–12. 10.1056/NEJMoa1204885 . 23614586

[32] Caster MM, Norris AH, Butao C, Carr Reese P, Chemey E, Phuka J, et al Assessing the acceptability, feasibility, and effectiveness of a tablet-based cervical cancer educational intervention. J Cancer Educ. 2017;32(1):35–42. Epub 2015/12/08. 10.1007/s13187-015-0953-6 26637473 PMC4894001

[33] Thys S, Mwape KE, Lefevre P, Dorny P, Marcotty T, Phiri AM, et al Why latrines are not used: communities' perceptions and practices regarding latrines in a *Taenia solium* endemic rural area in eastern Zambia. PLOS Negl Trop Dis. 2015;3 4, 2015 10.1371/journal.pntd.0003570 25739017 PMC4352092

[34] Bardosh K, Inthavong P, Xayaheuang S, Okello AL. Controlling parasites, understanding practices: the biosocial complexity of a One Health intervention for neglected zoonotic helminths in northern Lao PDR. Social Science and Medicine. 2014;120:215–23. 10.1016/j.socscimed.2014.09.030 . 25261615

[35] Hobbs EC, Mwape KE, Phiri AM, Mambwe M, Mambo R, Thys S, et al Perceptions and acceptability of piloted *Taenia solium* control and elimination interventions in two endemic communities in eastern Zambia. Transboundary and Emerging Diseases. 2019;00:1–13. 10.1111/tbed.13214 In press. 31231968 PMC7496623

[36] Lansdown R, Ledward A, Hall A, Issae W, Yona E, Matulu J, et al Schistosomiasis, helminth infection and health education in Tanzania: achieving behaviour change in primary schools. Health Educ Res. 2002;17(4):425–33. 10.1093/her/17.4.425 12197588

[37] Bamani S, Toubali E, Diarra S, Goita S, Berté Z, Coulibaly F, et al Enhancing community knowledge and health behaviors to eliminate blinding trachoma in Mali using radio messaging as a strategy. Health Educ Res. 2013;28(2):360–70. 10.1093/her/cys105 23125253

[38] Krecek, Krecek cc. Let's break the pork tapeworm cycle with these 6 easy steps. Nairobi, Kenya: International Livestock Research Institute and Medical Research Council of South Africa (ILRI); 2005.

