# Supplementary material for: Effects of ‘The Vicious Worm’ educational tool on Taenia solium knowledge retention in Zambian primary school students after one year
Source: PLoS Negl Trop Dis. 2019 May 20;13(5):e0007336. doi: 10.1371/journal.pntd.0007336 (PMC6544326; doi:10.1371/journal.pntd.0007336)
Supplement: S1 Table — a 'Masese' is the local (Chewa language) word for CC. ND = not done. (DOCX) [file pntd.0007336.s001.docx]

|  | | **INTIAL WORKSHOP** | | | **FOLLOW-UP SESSION** | | | | |
| --- | --- | --- | --- | --- | --- | --- | --- | --- | --- |
|  |  | **Correct during 'pre' (%)** | **Correct during 'post' (%)** | **Knowledge change (%)** | **Correct during 'follow-up' (%)** | **Knowledge change (%): 'Follow up' vs 'post'** | **P-value for difference: 'Follow up' vs 'post'** | **Knowledge change (%): 'Follow up' vs 'pre'** | **P-value for difference: 'Follow up' vs 'pre'** |
| Have you ever heard about *masese^a^?* | | 70.0 | 75.0 | 5.0 | 87.5 | 12.5 | ND | 17.5 | ND |
| **Category 1: Acquisition & transmission of *T. solium* infections** | | **65.3** | **85.0** | **19.7** | **82.3** | **-2.7** | **0.853** | **17.0** | **0.015** |
|  | How can a pig become infected with PCC? | 78.0 | 90.0 | 12.0 | 90.6 | 0.6 | ND | 12.6 | ND |
|  | How do people get *T. solium* tapeworm infection? | 50.0 | 85.0 | 35.0 | 75.0 | -10.0 | ND | 25.0 | ND |
|  | A person infected with *T. solium* tapeworm will shed many eggs through…? | 68.0 | 80.0 | 12.0 | 81.3 | 1.3 | ND | 13.3 | ND |
| **Category 2: Acquisition of NCC** | | **26.5** | **20.5** | **-6.0** | **53.1** | **32.6** | **<0.001** | **26.6** | **0.003** |
|  | A person with NCC/CC might have got the infection by…? | 25.0 | 13.0 | -12.0 | 40.6 | 27.6 | ND | 15.6 | ND |
|  | A person with NCC can transmit the disease to others through…? | 28.0 | 28.0 | 0.0 | 65.6 | 37.6 | ND | 37.6 | ND |
| **Category 3: TS in general** | | **74.3** | **85.3** | **11.0** | **89.6** | **4.3** | **0.580** | **15.3** | **0.015** |
|  | What is human tapeworm infection/TS? | 65.0 | 80.0 | 15.0 | 81.3 | 1.3 | ND | 16.3 | ND |
|  | How can human tapeworm infection/TS be diagnosed? | 75.0 | 88.0 | 13.0 | 96.9 | 8.9 | ND | 21.9 | ND |
|  | How can TS be treated? | 83.0 | 88.0 | 5.0 | 90.6 | 2.6 | ND | 7.6 | ND |
| **Category 4: NCC in general** | | **69.3** | **87.0** | **17.7** | **88.5** | **1.5** | **0.909** | **19.2** | **0.003** |
|  | What is human NCC? | 35.0 | 73.0 | 38.0 | 84.4 | 11.4 | ND | 49.4 | ND |
|  | What are the symptoms of NCC? | 100.0 | 98.0 | -2.0 | 100.0 | 2.0 | ND | 0.0 | ND |
|  | What should a person who experiences seizures/chronic headache do? | 73.0 | 90.0 | 17.0 | 81.3 | -8.8 | ND | 8.3 | ND |
| **Category 5: PCC diagnosis** | | **68.8** | **83.8** | **15.0** | **89.8** | **6.1** | **0.293** | **21.1** | **<0.001** |
|  | What is PCC? | 75.0 | 100.0 | 25.0 | 93.8 | -6.3 | ND | 18.8 | ND |
|  | What does PCC look like? | 60.0 | 85.0 | 25.0 | 93.8 | 8.8 | ND | 33.8 | ND |
|  | How can you test for PCC in a live pig? | 60.0 | 90.0 | 30.0 | 96.9 | 6.9 | ND | 36.9 | ND |
|  | How can PCC be diagnosed in a slaughtered pig? | 80.0 | 60.0 | -20.0 | 75.0 | 15.0 | ND | -5.0 | ND |
| **Category 6: PCC treatment** | | **35.0** | **41.5** | **6.5** | **42.2** | **0.7** | **0.993** | **7.2** | **0.652** |
|  | What should ideally be done with a live pig that has PCC? | 52.0 | 75.0 | 23.0 | 43.8 | -31.3 | ND | -8.3 | ND |
|  | What should be done with a slaughtered pig that has PCC? | 18.0 | 8.0 | -10.0 | 40.6 | 32.6 | ND | 22.6 | ND |
| **Category 7: Relationship between PCC/TS/NCC** | | **61.0** | **67.7** | **6.7** | **54.2** | **-13.5** | **0.114** | **-6.8** | **0.586** |
|  | Is PCC a problem for human health? | 88.0 | 88.0 | 0.0 | 56.3 | -31.8 | ND | -31.8 | ND |
|  | Are PCC and human tapeworm related? | 65.0 | 55.0 | -10.0 | 46.9 | -8.1 | ND | -18.1 | ND |
|  | What problems can an adult *T. solium* tapeworm cause? | 30.0 | 60.0 | 30.0 | 59.4 | -0.6 | ND | 29.4 | ND |
| **Category 8: Prevention of PCC/TS/NCC** | | **70.3** | **85.3** | **15.0** | **81.3** | **-4.1** | **0.668** | **10.9** | **0.143** |
|  | How can you prevent pigs getting PCC? | 93.0 | 98.0 | 5.0 | 96.9 | -1.1 | ND | 3.9 | ND |
|  | How can you prevent human tapeworm infections? | 52.0 | 70.0 | 18.0 | 65.6 | -4.4 | ND | 13.6 | ND |
|  | How can human CC/NCC be prevented? | 66.0 | 88.0 | 22.0 | 81.3 | -6.8 | ND | 15.3 | ND |
| **OVERALL QUESTIONNAIRE AVERAGES** | | **62.0** | **73.5** | **11.5** | **76.0** | **2.5** | **0.404** | **14.0** | **<0.001** |
