# Supplementary material for: Effects of ‘The Vicious Worm’ educational tool on Taenia solium knowledge retention in Zambian primary school students after one year
Source: PLoS Negl Trop Dis. 2019 May 20;13(5):e0007336. doi: 10.1371/journal.pntd.0007336 (PMC6544326; doi:10.1371/journal.pntd.0007336)
Supplement: S2 Table — a 'Masese' is the local (Chewa language) word for CC. ND = not done. (DOCX) [file pntd.0007336.s002.docx]

|  | | **INITIAL WORKSHOP** | | | **FOLLOW-UP SESSION** | | | | |
| --- | --- | --- | --- | --- | --- | --- | --- | --- | --- |
|  | | **Correct during 'pre' (%)** | **Correct during 'post' (%)** | **Knowledge change (%)** | **Correct during 'follow up' (%)** | **Knowledge change (%): 'Follow up' vs 'Post'** | **P-value for difference: 'Follow up' vs 'post'** | **Knowledge change (%): 'Follow up' vs 'Pre'** | **P-value for difference: 'Follow up' vs 'pre'** |
| **Category 1: General knowledge** | | **70.8** | **90.8** | **20.0** | **75.9** | **-14.9** | **<0.001** | **5.1** | **0.105** |
|  | Have you ever heard of *masese^a^*? | 76.3 | 96.6 | 20.3 | 90.7 | -5.9 | ND | 14.5 | ND |
|  | What is PCC? | 84.7 | 100.0 | 15.3 | 81.5 | -18.5 | ND | -3.3 | ND |
|  | What does PCC look like in pigs? | 74.6 | 91.5 | 16.9 | 87.0 | -4.5 | ND | 12.5 | ND |
|  | What is human tapeworm/TS? | 59.3 | 72.9 | 13.6 | 50.0 | -22.9 | ND | -9.3 | ND |
|  | What are the symptoms of NCC? | 59.3 | 93.2 | 33.9 | 70.4 | -22.8 | ND | 11.1 | ND |
| **Category 2: Transmission** | | **48.1** | **68.8** | **20.7** | **59.6** | **-9.2** | **0.058** | **11.5** | **0.035** |
|  | How can a pig become infected with PCC? | 33.9 | 91.5 | 57.6 | 61.1 | -30.4 | ND | 27.2 | ND |
|  | How do people get TS? | 78.0 | 100.0 | 22.0 | 83.3 | -16.7 | ND | 5.3 | ND |
|  | How does a person with TS shed eggs into the environment? | 49.2 | 86.4 | 37.3 | 77.8 | -8.6 | ND | 28.6 | ND |
|  | How do people get CC? | 33.9 | 32.2 | -1.7 | 27.8 | -4.4 | ND | -6.1 | ND |
|  | Can people with NCC transmit it to others? | 45.8 | 33.9 | -11.9 | 48.1 | 14.2 | ND | 2.3 | ND |
| **Category 3: Prevention** | | **65.8** | **86.4** | **20.7** | **78.1** | **-8.3** | **0.020** | **12.3** | **0.013** |
|  | Can you eat the meat of a slaughtered pig with PCC? | 49.2 | 89.8 | 40.7 | 77.8 | -12.0 | ND | 28.6 | ND |
|  | Can you prevent pigs getting PCC? | 44.1 | 86.4 | 42.4 | 75.9 | -10.5 | ND | 31.8 | ND |
|  | How can TS be treated? | 100.0 | 98.3 | -1.7 | 92.6 | -5.7 | ND | -7.4 | ND |
|  | How can you prevent TS? | 81.4 | 96.6 | 15.3 | 81.5 | -15.1 | ND | 0.1 | ND |
|  | How can CC and NCC be prevented? | 54.2 | 61.0 | 6.8 | 63.0 | 1.9 | ND | 8.7 | ND |
| **OVERALL QUESTIONNAIRE AVERAGES** | | **61.6** | **82.0** | **20.4** | **71.2** | **-10.8** | **<0.001** | **9.6** | **<0.001** |
