# Supplementary figures and images for: Effects of ‘The Vicious Worm’ educational tool on Taenia solium knowledge retention in Zambian primary school students after one year
Source: PLoS Negl Trop Dis. 2019 May 20;13(5):e0007336. doi: 10.1371/journal.pntd.0007336 (PMC6544326; doi:10.1371/journal.pntd.0007336)

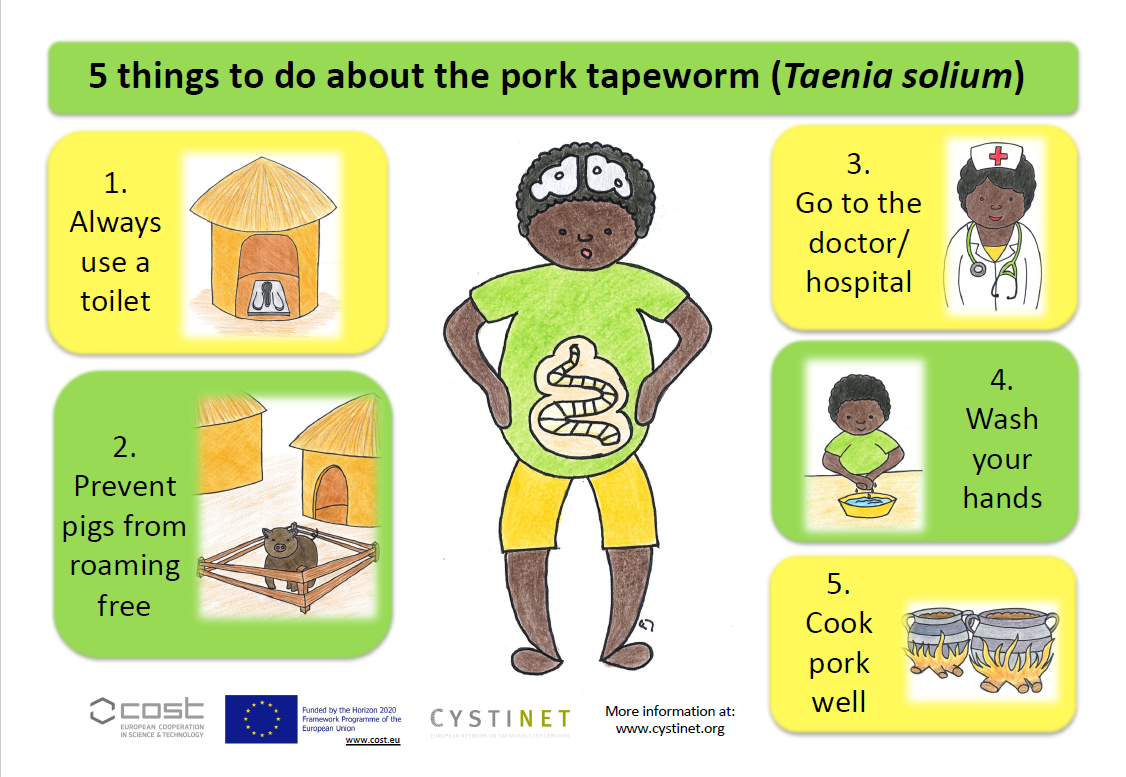
a)


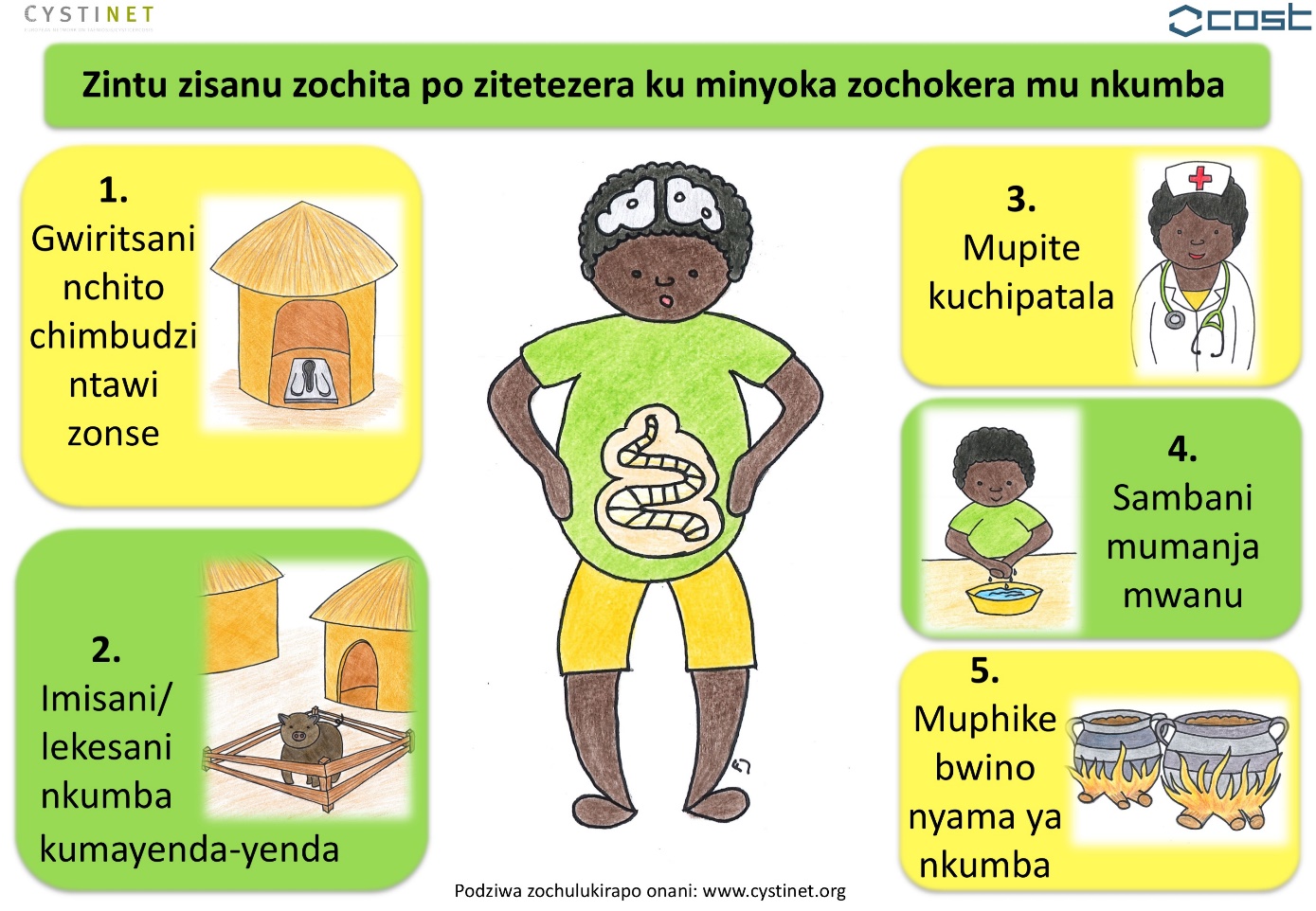
b)

Supplement: S1 File — Educational posters: ‘5 things to do about the pork tapeworm (Taenia solium)’, in English (a) and Chewa (b). Developed by CYSTINET (http://www.cystinet.org/). Illustrations by F. Jansen. (DOCX) [file pntd.0007336.s003.docx]
